# Metabolic plasticity drives specific mechanisms of chemotherapy and targeted therapy resistance in metastatic colorectal cancer

**DOI:** 10.37349/etat.2025.1002337

**Published:** 2025-09-23

**Authors:** Mariam Rojas, Malena Manzi, Sergio Madurga, Fernando Enrique García Velásquez, Maira Alejandra Romero, Silvia Marín, Marta Cascante, Joan Maurel

**Affiliations:** University of Catania, Italy; ^1^Medical Oncology Department, Hospital Clínic of Barcelona, Translational Genomics and Targeted Therapeutics in Solid Tumors Group, Institut d’Investigacions Biomèdiques August Pi i Sunyer (IDIBAPS), University of Barcelona, 08036 Barcelona, Spain; ^2^CIBEREHD, Network Centre for Hepatic and Digestive Diseases, National Spanish Health Institute Carlos III (ISCIII), 28029 Madrid, Spain; ^3^Department of Biochemistry and Molecular Biomedicine, Institute of Biomedicine (IBUB), Universitat de Barcelona, 08036 Barcelona, Spain

**Keywords:** metabolic subtypes, IMMETCOLS, chemotherapy resistance, targeted therapy, colorectal cancer

## Abstract

Microsatellite-stable metastatic colorectal cancer (MSS mCRC) is currently treated with chemotherapy and targeted agents based on RAS and BRAF mutational status. Although these therapies offer initial benefit, most patients rapidly develop resistance, with fewer than 20% remaining progression-free at two years. This review aims to synthesize emerging evidence on the metabolic mechanisms driving treatment resistance in MSS mCRC, with a particular focus on the immune-metabolic signature (IMMETCOLS) classification. We conducted a comprehensive review of preclinical models, transcriptomic datasets, and clinical trial results addressing metabolic adaptations to chemotherapy and targeted therapies in MSS mCRC. The IMMETCOLS framework defines three metabolic subtypes—IMC1, IMC2, and IMC3—each associated with distinct resistance mechanisms. IMC1 exhibits glycolysis and transforming growth factor-β (TGF-β)-dependent signaling enriched in inflammatory fibroblasts, conferring resistance to chemotherapy. IMC2 relies on oxidative phosphorylation and glutamine metabolism, supporting antioxidant defenses and resistance to both cytotoxic agents and anti-EGFR therapies. IMC3 demonstrates lactate-fueled respiration and pentose phosphate pathway activation, contributing to redox balance, DNA repair, and resistance to targeted therapies such as anti-BRAF or KRAS inhibitors. All subtypes display metabolic plasticity under therapeutic pressure. Emerging clinical data support tailoring targeted therapy combinations based on IMMETCOLS subtype, particularly in BRAF- and HER2-positive populations. Understanding subtype-specific metabolic rewiring in MSS mCRC offers novel opportunities to overcome drug resistance. Targeting the metabolic vulnerabilities defined by the IMMETCOLS signature may improve response durability and inform precision treatment strategies.

## Introduction

Our group has defined an immune-metabolic signature (IMMETCOLS) in metastatic colorectal cancer (mCRC) that is conserved across diverse tumor types. This classification integrates clinical presentation, the immune and stromal microenvironment, and complex metabolic pathway analysis [1, 2]. Three cluster subtypes have been identified: (i) the IMC1 subtype, a mesenchymal subtype characterized by atypical glycolysis (Warburg effect) and low oxidative phosphorylation (OXPHOS), which constitutes 20–36% of all patients; (ii) IMC2, an epithelial subtype with glycolysis and glutaminolysis and intermediate OXPHOS, which represents 13–19% of patients; and (iii) IMC3, an epithelial subtype with concomitant high glycolysis and high OXPHOS, which constitutes 49–63% of patients.

We have also employed the IMMETCOLS signature to assess the immunometabolic characteristics of consensus molecular subtypes (CMS) in CRC, drawing on retrospective data from stage I–IV CRC patients and leveraging public datasets such as TCGA and GES1 [2]. Patients classified as CMS4 predominantly fall into the IMC1 category (90%), with both CMS4 and IMC1 being associated with a poor prognosis. CMS1 is characterized by a mixture of IMC1 (47%) and IMC3 (47%), whereas CMS2 and CMS3 subtypes are mainly found in IMC3 (71%) and IMC2 (22%) clusters. Despite linking metabolic signatures with immune infiltration patterns, the IMMETCOLS classification lacks detailed metabolic information on each cellular component (fibroblasts, immune cells, and cancer cells) and is a static signature. The aim of this review is to describe the metabolic mechanisms that enable tumors to evade treatment efficacy. The insights gained may facilitate the development of novel approaches to overcome drug resistance and support new cancer treatment strategies.

## CRC omics classification

The CMS classification is the most widely studied transcriptomic classification in CRC. It distinguishes four subtypes: CMS1 (microsatellite instability/immune, 14%), CMS2 (canonical, 37%), CMS3 (metabolic, 13%), and CMS4 (mesenchymal, 23%). CMS1 is associated with poor prognosis in advanced disease, whereas CMS4 is linked to poor prognosis in localized disease [3]. However, despite its widespread adoption, this classification system does not adequately capture the metabolic characteristics of each subtype and offers limited value for clinical decision-making. Because the CMS subtype classification was developed using bulk transcriptomic data, other researchers have re-evaluated it using single-cell RNA (scRNA) analysis. A carefully constructed study by Joanito et al. [4] reclassified CRC patients based solely on scRNA analysis of malignant cells into two intrinsic (i) subtypes: iCMS2 and iCMS3. Interestingly, recapitulated bulk transcriptomics revealed that the iCMS3 subtype encompasses both *BRAF* mutant and microsatellite-unstable (MSI) patients. In contrast, iCMS2 tumors were predominantly microsatellite-stable (MSS), left-sided, and characterized by high MYC and WNT pathway activity, frequent APC and TP53 mutations, low immune infiltration, and gene expression signatures associated with increased sensitivity to chemotherapy and anti-EGFR therapy.

Recently, a new transcriptomic classification distinguished three different subtypes based on pathway-derived subtype (PDS) [5]. PDS1 is a canonical subtype, enriched in MYC and E2F targets, G2M checkpoint, and OXPHOS pathways, and is associated with a good prognosis. PDS2 is characterized by stromal and immune microenvironment enrichment. PDS3 mainly comprises CMS2 and CMS3 subtypes. While PDS3 is associated with poor prognosis, the analysis was conducted exclusively in CRC cohorts with limited disease, leaving the significance of PDS3 in advanced disease and its correlation with clinical and metabolic features currently unknown.

Colon cancer subtypes have also been investigated using comprehensive multi-omics approaches, encompassing genomic, transcriptomic, proteomic, and phospho-proteomic analyses. For example, Vasaikar et al. [6] analyzed MSI and MSS patients, differentiating three subtypes: MSI, mesenchymal (about one-third of MSS patients), and chromosomal instability (CIN; about two-thirds of MSS patients). Interestingly, compared with MSS patients, MSI patients show increased glycolysis and decreased tricarboxylic acid cycle (TCA) activity at the protein level but not at the transcriptomic level. Another multi-omics study indicated that a particular subset of MSS patients, associated with poor prognosis, exhibited increased protein expression related to the citrate cycle, OXPHOS, glycolysis, and fatty acid synthesis [7]. This poor-prognosis group, which demonstrates unfavorable outcomes in mCRC, primarily comprises the CMS2 and CMS3 subtypes. This suggests that distinct metabolic profiles with potential clinical implications may coexist within these CMS subtypes.

## Transcriptomic classifications based on metastatic samples from CRC

Several research groups have evaluated CMS subtypes in CRC patients who have undergone cytoreductive surgery, identifying CMS4 as the most abundant phenotype. CMS concordance between primary tumors and metastases was found in between 63% and 92% of cases [8, 9]. Importantly, Lenos et al. [9] showed that there are specific subtypes within CMS4, identifying one subtype characterized by DNA replication and E2F targets that exhibited a higher peritoneal cancer index and worse prognosis. In addition, several studies have evaluated CMS subtype distributions using biopsies obtained from resected liver and lung metastases [10–13]. Kamal et al. [11] identified two main metastasis groups based on transcriptomic features. The first group—M1—was primarily characterized by inflammation featuring adaptive immune system responses and immune evasion pathways (e.g., PD-1 signaling and lymphocytic cell-mediated immunity). The second group—M2—was characterized by cell proliferation and MYC signaling. Notably, epithelial-mesenchymal transition (EMT) activity was enriched in both the M1 cluster and post-treatment metastases, while MYC activity was more prominent in pretreatment metastases and the M2 cluster, suggesting that these metastatic phenotypes may be influenced by treatment exposure. Overall, most tumor biopsies from metastases belong to CMS2 and CMS4 subtypes, with very few patients classified as CMS1 or CMS3, and an increased proportion of CMS2 compared with CMS4 in liver metastases.

As reported by Guinney et al. [3], the CMS1 subtype showed the worst prognosis in the largest studies published by Piskol et al. [10], Eide et al. [12], and Chowdhury et al. [13]. In resected liver metastases, two studies reported that the canonical subtype (mainly characterized by CMS2) shows the worst prognosis [14, 15]. We have to note that all of these studies included patients with oligometastatic disease only. Therefore, the CMS distribution in more bulky metastatic disease is currently unknown. In addition, most of these studies lack critical clinical information (including ECOG performance status), details of disease extent (such as the organs involved), and biochemical parameters related to tumor biology (including at least lactate dehydrogenase and C-reactive protein), which are essential for unbiased survival analysis. These distinct metabolic signatures and their corresponding vulnerabilities are summarized in Table 1.

**Table 1:** Transcriptomic characteristics in metastatic biopsies.

**References**	**M1 location**	**Bulk transcriptomics**	**Concordance (primary and M1)**
Laoukili et al. [8] (*n* = 12)	Peritoneal*, &	CMS4 (100%)	92%
Lenos et al. [9] (*n* = 52)	Peritoneal**, &	CMS1 (6%), CMS2 (8%), CMS3 (2%), CMS4 (85%)	63%
Piskol et al. [10] (*n* = 130)	Not defined&	CMS1 (17%), CMS2 (48%), CMS3 (8%), CMS4 (27%)	60%
Kamal et al. [11] (*n* = 257)	Lung, liver*	M1 (86%) and CMS4 (85%); M2 (63%) and CMS2 (52%)	NE
Eide et al. [12] (*n* = 295)	Liver*, &	CMS1 (6%), CMS2 (35%), CMS3 (2%), CMS4 (35%)	33%
Chowdhury et al. [13] (*n* = 10,776)	Multiple sites	CMS1 (10–16%), CMS2 (37–45%), CMS3 (11–13%), CMS4 (27–39%)	NE
Pitroda et al. [14] (*n* = 134)	Liver**, &	Canonical (33%), immune (28%), stromal (39%)	NE
Katipally et al. [15] (*n* = 240)	Liver**, &	Canonical (50%), immune (19%), stromal (31%)	NE

*: Sample obtained from untreated patients; **: sample obtained from pretreated patients; &: sample obtained from resected metastatic biopsies; *n*: number of patients included in each cohort; M1: transcriptomic subtype characterized by inflammatory and T-cell infiltration; M2: transcriptomic subtype enriched in MYC and E2F targets. CMS: consensus molecular subtypes; NE: not evaluated.

## Metabolic adaptation to chemotherapy in mCRC

According to the IMMETCOLS classification, IMC1 is defined as a glycolytic subtype enriched in stromal components, transforming growth factor-β (TGF-β) signaling, extracellular matrix interactions, collagen synthesis, and the hexosamine biosynthesis pathway. It is further characterized by an inflamed tumor microenvironment. This specific subtype is supported by diverse cancer-associated fibroblast (CAF)-cancer cell interactions and specific metabolic dependencies [16–22]. Two types of CAFs have been identified: inflammatory CAFs (iCAFs) and myofibroblast CAFs (mCAFs). iCAFs secrete IL-6, IL-1, IL-11, and leukemia inhibitory factor (LIF) and exhibit a loss of myofibroblastic features. In contrast, mCAFs are characterized by elevated expression of α-smooth muscle actin (α-SMA) and TGF-β [16]. These two CAF subtypes are regulated by metabolic interactions. mCAFs utilize a TGF-β-mediated mitochondrial oxidation of glucose and glutamine to support collagen synthesis [17]. Conversely, iCAFs, in a TGF-β-independent manner, use extracellular lactate to replenish the TCA cycle and support collagen synthesis when glutamine and glucose are limited [18]. Importantly, both TGF-β-dependent pathways (which rely on glucose and glutamine) and TGF-β-independent pathways (which utilize lactate) converge to enhance mitochondrial metabolism, leading to increased production of damaging reactive oxygen species (ROS) and a reduced ability to generate proline and collagen as a protective vent.

Beyond CAFs, immune cells like TAMs, Tregs, and myeloid-derived suppressor cells (MDSCs) can shape tumor metabolism by competing for nutrients and releasing metabolites that reinforce the IMC1 phenotype and therapy resistance.

Currently, it is unclear which of these specific CAFs, if any, are related to chemotherapy resistance in clinical settings. In rectal cancer, for instance, iCAFs and IL-1 have been associated with chemoradiotherapy resistance [19], as has a fibroblast-TGF-β signature [20]. This contradiction is also evident in preclinical studies, where iCAFs (e.g., CD10^+^GPR77^+^) have been shown to support chemoresistance and cancer stemness through NF-κB activation [21]. In addition, mCAFs have been demonstrated to promote chemoresistance in CRC through TGF-β2 and hypoxia [22]. Hypoxia is a key regulator of metabolic plasticity, promoting adaptive responses such as the shift from oxidative to reductive glutamine metabolism, enhanced fatty acid oxidation, and lactate utilization. Moreover, ZEB1, a master regulator of EMT, has been found to coordinate meiotic recombination 11 homolog (MRE11) and chemoresistance [23]. Epigenetic changes, including histone modifications and lactate-driven lactylation, also contribute to resistance. For example, lactate-driven lactylation, a product of the Warburg effect, facilitates the MRE11-RAD50-NBS complex, enhancing homologous recombination repair and chemoresistance [24, 25]. Evidence from clinical trials indicates that targeting the IMC1 subtype remains challenging. Ongoing trials with NIS793 (a TGF-β inhibitor) and M7824 (a PD-L1/TGF-β dual inhibitor) are currently evaluating efficacy in all mCRC patients.

IMC2 primarily relies on OXPHOS and the consumption of glucose and glutamine. Key metabolic mechanisms of chemoresistance in the IMC2 subtype include enhanced fatty acid oxidation, glutamine reductive carboxylation, and increases in polyamine synthesis and concomitant antioxidant processes [26–29]. Pretreatment, these tumors can utilize glutamine in two different ways: they can oxidize glutamine through glutamine dehydrogenase (glutamine anaplerosis) to enter the TCA cycle or derive glutamine through transamination to increase OXPHOS, enhance antioxidant processes (mainly increasing ferroptosis resistance), and support fatty acid synthesis. It is unknown which of these two glutamine uses, if either, is related to intrinsic chemotherapy resistance.

**Figure 1:**
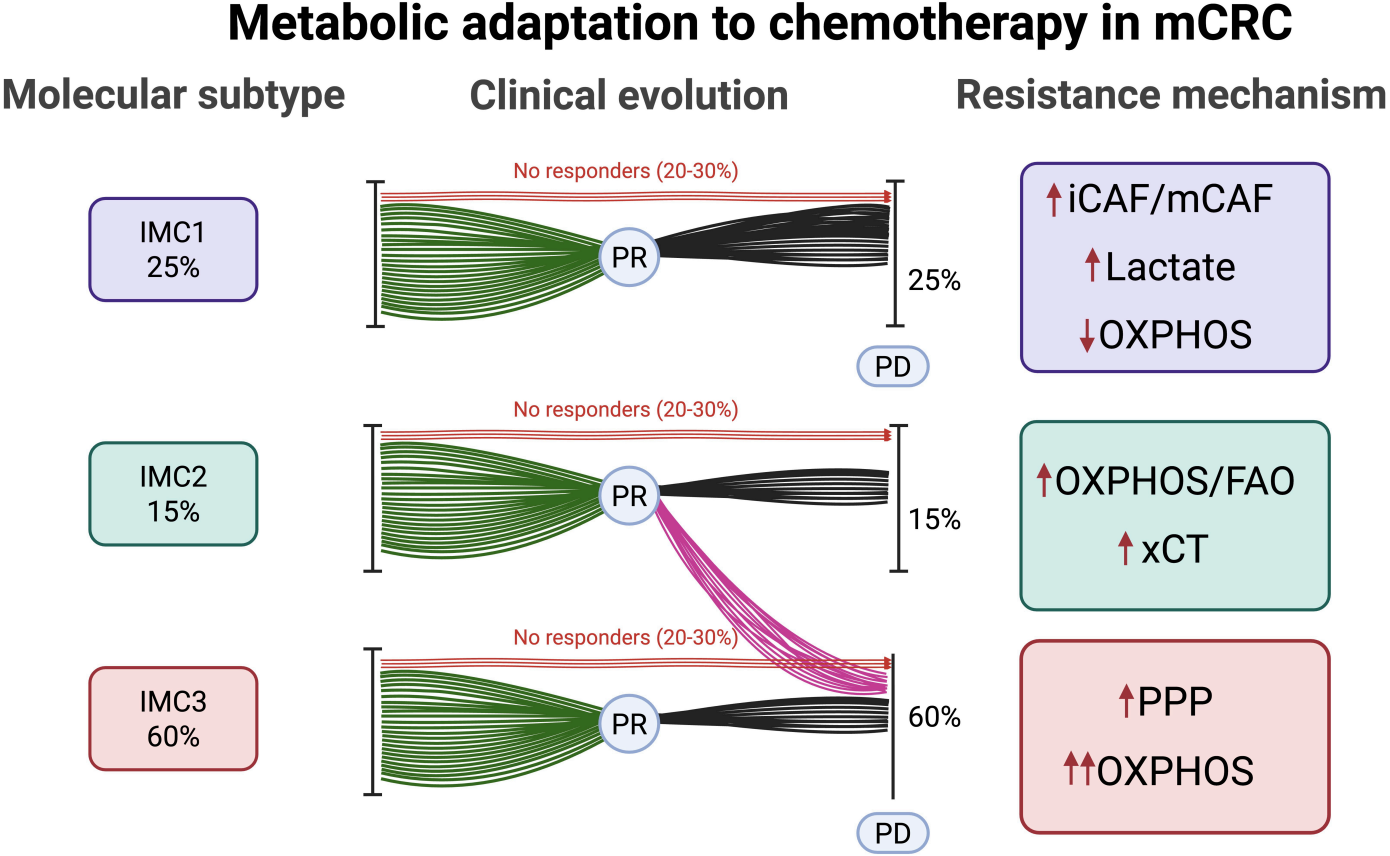
**Distribution, clinical evolution, and acquired resistance mechanisms across IMMETCOLS subtypes.** IMC1 (25%): mesenchymal, low OXPHOS, resistance via lactate secretion and CAF-driven signaling (iCAF/mCAF). IMC2 (15%): epithelial, resistance via FAO, OXPHOS, and xCT-mediated antioxidant defense. IMC3 (60%): epithelial, resistance via PPP activation (G6PD/PLK1 axis) and enhanced OXPHOS. OXPHOS: oxidative phosphorylation; FAO: fatty acid oxidation; xCT: cystine/glutamate antiporter; PPP: pentose phosphate pathway; G6PD: glucose-6-phosphate dehydrogenase; PLK1: polo-like kinase 1; IMMETCOLS: immune-metabolic signature; PR: partial response; PD: progressive disease; CAF: cancer-associated fibroblast; iCAF/mCAF: inflammatory CAF/myofibroblast CAF; mCRC: metastatic colorectal cancer. Created in BioRender. Maurel, J. (2025) https://BioRender.com/s9ph5vb.

After chemotherapy exposure, enhanced OXPHOS has also been identified as a hallmark of chemoresistance. CRC cells increase mitochondrial respiration through the sirtuin 1 (SIRT1)/peroxisome proliferator-activated receptor gamma coactivator 1-alpha (PGC1α) axis in response to chemotherapy exposure, favoring survival [30]. This adaptive shift from glycolysis to increased OXPHOS through fatty acid oxidation is a key trait of IMC2 tumors. IMC2 tumors resist ferroptosis, a form of iron-dependent lipid peroxidation-driven cell death, by increasing the antiporter SLC7A11 [cystine/glutamate antiporter (xCT)], which exchanges intracellular glutamate for extracellular cystine, supporting a high glutathione (GSH)/GSSG ratio, and enhancing antioxidant protection. Multiple tumor types exposed to different therapeutic agents have been shown to develop this mechanism of acquired resistance. Examples include tumors treated with paclitaxel [31–33] or docetaxel [34], triple-negative breast cancer (TNBC) treated with doxorubicin combined with cyclophosphamide [35], gastrointestinal tumors exposed to oxaliplatin [36], squamous esophageal cancer treated with cisplatin [37], and glioblastoma [38] and lung cancer [39] treated with radiotherapy. Therefore, we conclude that this is a common rewiring mechanism employed by various solid tumors under the selective pressure of chemotherapy and radiotherapy that enables their survival when the glucose supply is diminished. Moreover, we cannot rule out the possibility that other, more glycolytic tumors, such as the IMC3 subtype, also use this metabolic rewiring process to survive in a latent state at the time of maximum response (see Figure 1).

Another interesting potential mechanism of acquired resistance in the IMC2 subtype is related to polyamine metabolism. In TNBC, acquired chemoresistance has been linked to ornithine decarboxylase 1 (ODC1), a rate-limiting enzyme of polyamine synthesis. Targeting ODC1 sensitizes TNBC cells to chemotherapy [40]. In addition, spermidine/spermine N1-acetyltransferase (SAT1), a rate-limiting enzyme of polyamine catabolism, has been associated with chemotherapy resistance in pancreatic cancer [41]. SAT1 promotes OXPHOS and simultaneously activates antioxidant mechanisms through a glutamine rewiring process, enhancing the conversion of glutamine to glutamate and subsequently boosting GSH synthesis [42]. Although OXPHOS inhibition has demonstrated efficacy in preclinical settings [43–45], alternative nutrients such as glutamine [46] and lactate [47] can fuel mitochondrial respiration. Accordingly, an OXPHOS inhibitor, IACS-010759, has recently been tested in the clinic in solid tumors but showed very limited activity and high toxicity [48].

The IMC3 subtype is characterized by mitochondrial lactate uptake, a high TCA flux, and a high OXPHOS metabolic profile that facilitates, in mCRC, extensive liver metastases and a high lactate dehydrogenase level at disease presentation [49–52]. How these tumors, initially extremely sensitive to current first-line therapies, rapidly acquire resistance is currently unknown. Because these tumors persist with high glycolysis (but not the Warburg effect) at disease progression, we hypothesize that they utilize atypical glycolytic pathways [mainly the oxidative and non-oxidative pentose phosphate pathway (PPP)] to sustain the elevated TCA flux/OXPHOS and drive acquired resistance. For instance, high levels of glucose-6-phosphate dehydrogenase (G6PD) and polo-like kinase 1 (PLK1) expression have been related to chemotherapy resistance in CRC [53, 54]. Because PLK1 activates PPP by direct G6PD phosphorylation [55], inhibiting PLK1 may be an interesting strategy to overcome acquired resistance. Although PLK1 is a crucial participant in this repair process, it is unclear how PLK1 functions after therapy. For instance, PLK1 stabilization through ubiquitin-binding protein 2-like depletion leads to severe mitotic defects [56]. Therefore, nonfunctional PLK1 may simply allow cells with unrepaired double-strand breaks to enter the cell cycle, increasing genome instability [57, 58]. Interestingly, preclinical phosphogluconate dehydrogenase inhibition increases the activation of alternative metabolic pathways such as glutamine reductive carboxylation [59] and glutaminolysis [60, 61]. Although no trials have yet included patients based on IMC3, agents targeting relevant pathways are under investigation. Notably, the PLK1 inhibitor onvansertib has been evaluated in second-line therapy with FOLFIRI and bevacizumab in *KRAS*-mutant mCRC. An overall response rate of 26.4% and an impressive objective response rate of 76.9% were observed in bevacizumab-naïve patients [62].

**Figure 2:**
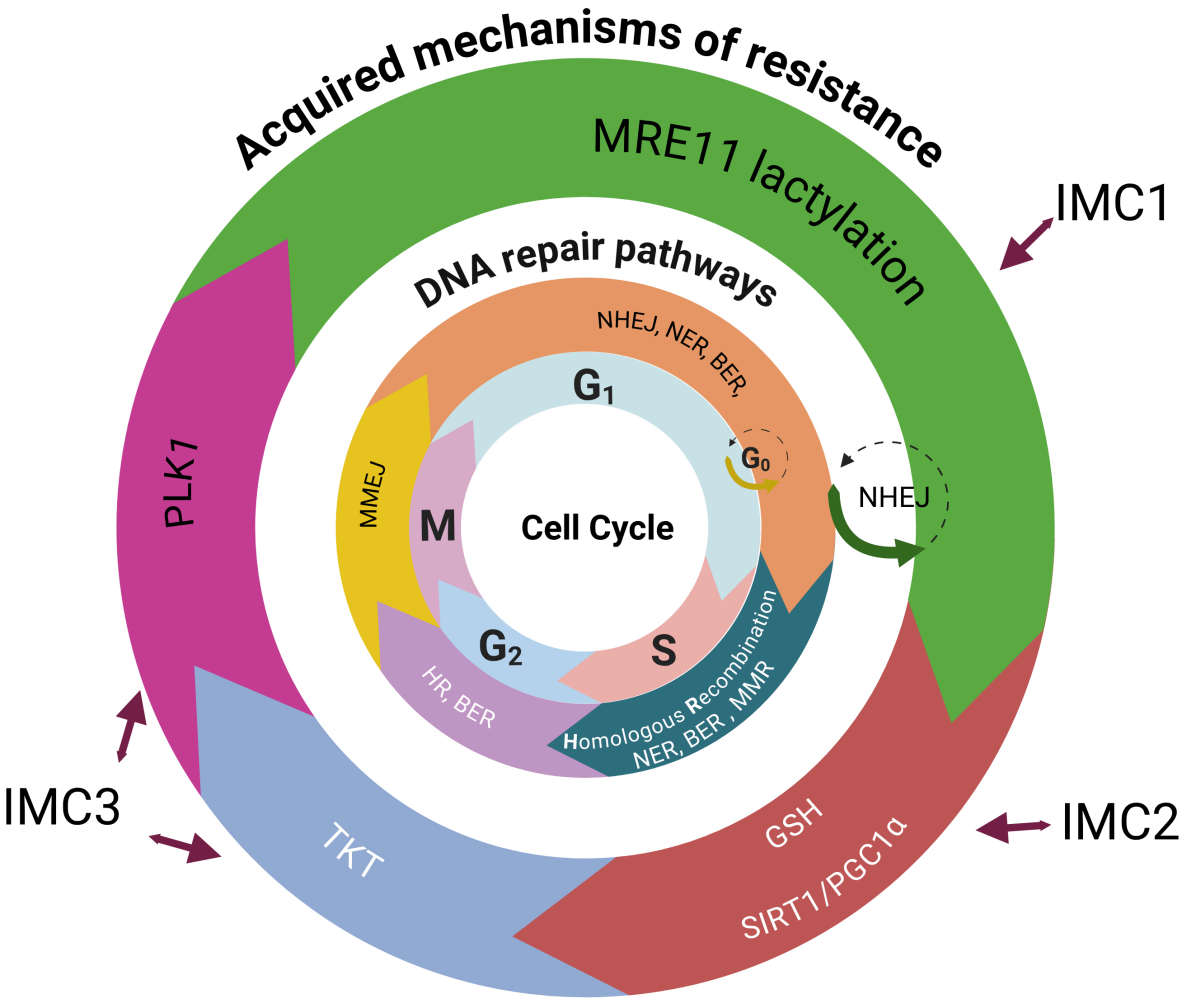
**Acquired resistance mechanisms distributed by the DNA repair pathway and cell cycle phase across IMMETCOLS subtypes.** The figure illustrates how specific mechanisms of acquired resistance operate through distinct DNA repair pathways at defined phases of the cell cycle, in relation to IMMETCOLS subtypes. IMC1 tumors (20–36%) show aberrant homologous recombination; activation through MRE11 lactylation. IMC2 tumors (13–19%) rely on mitochondrial upregulation via the SIRT1/PGC1α axis and antioxidant defense mediated by GSH. IMC3 tumors (49–63%) adapt by enhancing the pentose phosphate pathway (PPP) and OXPHOS via PLK1 and TKT, promoting mitotic progression and DNA repair. IMMETCOLS: immune-metabolic signature; MRE11: meiotic recombination 11 homolog; HR: hazard ratio; SIRT1: sirtuin 1; PGC1α: peroxisome proliferator-activated receptor gamma coactivator 1-alpha; GSH: glutathione; OXPHOS: oxidative phosphorylation; PLK1: polo-like kinase 1; TKT: transketolase; NHEJ: non-homologous end joining; NER: nucleotide excision repair; BER: base excision repair; MMR: mismatch repair; MMEJ: microhomology-mediated end joining. Created in BioRender. Maurel, J. (2025) https://BioRender.com/9oo6vr3.

SIRT5, a member of the NAD^+^-dependent class III histone deacetylase family that activates transketolase (TKT), plays a role in CRC chemoresistance [63]. TKT plays a crucial role in double-strand break repair, and its depletion significantly reduces both non-homologous end joining and homologous recombination-mediated double-strand break repair. Mechanistically, TKT interacts with PARP1 and induces radioresistance in hepatocarcinoma [64]. In addition, TKT induces chemoresistance in glioma by translocating to the nucleus and interacting with XRN2 to remove R-loops [65]. Recent studies have shown that the product of TKTL1 fermentation metabolism, lactate, leads to lactylation and activation of MRE11, an enzyme that facilitates the repair of DNA strand breaks via homologous recombination [24, 25]. Therefore, in this specific subtype, the combination of a PLK1 inhibitor such as onvansertib with a TKT inhibitor such as benfo-oxythiamine might be beneficial in heavily pretreated mCRC patients [66]. In Figure 2, we have stated potential DNA repair mechanisms related to each of IMMETCOLS subtypes.

Although IMMETCOLS is a static signature based on primary tumor transcriptomics, extensive metabolic plasticity has been observed across all three subtypes. For instance, IMC2 tumors may adopt OXPHOS-related traits post-chemotherapy through fatty acid oxidation and glutamine rewiring, while IMC3 tumors rapidly acquire resistance via PPP activation and DNA repair enhancement. Similarly, anti-EGFR therapy has been shown to induce EMT-like features associated with IMC1, as discussed later. However, no current studies have demonstrated temporal transitions between subtypes, and IMMETCOLS has not been validated in liquid biopsies. While metabolic PET imaging could offer non-invasive monitoring of metabolic changes, its correlation with IMMETCOLS subtypes remains unexplored.

## Targeted therapy in mCRC

Table 2 presents the activity of upfront anti-EGFR therapy in combination with chemotherapy doublets in *RAS*/*BRAF* wild-type patients, who constitute roughly 30–35% of all mCRC patients. Currently, the best overall response (BOR) ranges between 67% and 89%, with a median progression-free survival (mPFS) of 12 months, but fewer than 20% of patients remain progression-free at 2 years. Therefore, despite high activity, particularly in left-sided tumors [67], acquired resistance is common. In *BRAF* mutant patients (8–10% of mCRC patients), randomized trials have demonstrated that doublet therapy inhibition (cetuximab combined with encorafenib or vemurafenib) in second-line therapy achieves a BOR of between 17% and 21%, which is clearly superior to that of irinotecan with cetuximab (< 5% activity) [68, 69]. In untreated *BRAF* mutant patients, the BREAKWATER trial showed an impressive BOR of 65.7% with FOLFOX-encorafenib and cetuximab vs. 37.4% with standard of care (SOC) FOLFOX. This resulted in an odds ratio of 2.44 [95% confidence interval (CI) 1.4–4.25], a median PFS of 12.8 months vs. 7.1 months [hazard ratio (HR) of 0.53; 95% CI 0.41–0.68], and a median overall survival of 30.3 months vs. 15.1 months (HR 0.49; 95% CI 0.38–0.63) [70, 71]. Mature long-term PFS and overall survival data are eagerly awaited.

**Table 2:** Intrinsic (basal) targeted therapy biomarkers of efficacy in metastatic colorectal cancer.

**References**	**Schedule of therapy**	**BOR%**	**HR (95% CI)**	**mPFS (95% CI)**	**12 months PFR%**
Shitara et al. [67] +	FOLFOX-PAN vs. FOLFOX-BEV	83.3 vs. 66.5*	0.76 (0.61–0.95)**	-	NE
Stintzing et al. [80] +	FOLFIRI-CET vs. FOLFIRI-BEV	88 vs. 71*** 76 vs. 55****	1.04 (0.73–1.43)***** 0.67 (0.45–0.99)*****	-	NE
Lenz et al. [81] +	FOLFOX/FOLFIRI-CET vs. FOLFOX-FOLFIRI-BEV	NE	0.91(0.62–1.23)***** 0.8% (0.68–1.21)*****	-	NE
Elez et al. [71] ++	FOLFOX-ENCO-CET vs. FOLFOX +/– BEV	65.1 vs. 37.4	0.53 (0.41–0.68)	-	
Middleton et al. [82] ++	DABRAFENIB-TRAMETINIB-PAN	38 vs. 7&&	4.33&& (*p* = 0.0012)	-	20 vs. < 5
Kopetz et al. [69] ++	IRI-VEMURAFENIB-CET vs. IRI-CET	17 vs. 4	0.3 vs. 0.6&&&	-	NE
Kopetz et al. [79] ++	ENCO-BINIMETINIB-CET vs. ENCO-CET vs. IRI-CET	26.8 vs. 19.5 vs. 1.8	1.85 (1.20–2.84)&&&& 0.56 (0.37–0.84)&&&&&	-	NE
Desai et al. [83] +++	DIVARASIB-CET	62.5	-	8.1 (5.5–12.3)$	< 15
Yaeger et al. [72] +++	ADAGRASIB-CET	34	-	6.9 (5.7–7.4)$	< 20
Fakih et al. [73] +++	SOTO (960)-PAN vs. SOTO (240)-PAN vs. SOC	26.4 vs. 5.7 vs. 0	0.49 (0.3–0.8) and 0.58 (0.36–0.93)$$		30 vs. 15 vs. 15
Siena et al. [75] ++++	TRASTUZUMAB DER	45.3	-	6.9 (4.1–NE)$	NE
Raghav et al. [76] ++++	TRASTUZUMAB DER (5.4) TRASTUZUMAB DER (6.4)	37.8 27.5	-	5.8 (4.6–7)$ 5.5 (4.2–7)$	< 10 < 10
Strickler et al. [77] ++++	TRASTUZUMAB + TUCATINIB	42.9	-	8.2 (4.2–10.3)$	34

+: RAS WT population; ++: BRAF mutant population; +++: KRAS G12C population; ++++: HER2-positive population. *: BOR comparing FOLFOX-PAN vs. FOLFOX-BEV in ctDNA left-sided double WT patients; **: HR for overall survival comparing FOLFOX-PAN vs. FOLFOX-BEV in left-sided double WT patients; ***: BOR in CMS2 comparing CET vs. BEV; ****: BOR in CMS4 comparing CET vs. BEV; *****: PFS HR between PAN/CET and BEV in CMS2 and CMS4 subtypes. &&: BOR comparing BM1 vs. BM2; &&&: HR for PFS benefit in BM1 vs. BM2 with doublets vs. SOC (HR extracted from [69]); &&&&: benefit in OS with double therapy in cytolytic-low (BM2); &&&&&: benefit in OS with triplet therapy in cytolytic-high (BM1). $: Median PFS and 95% CI; $$: HR for PFS comparing SOTO (960 mg) + PAN vs. SOTO (240 mg) + PAN vs. SC. BOR: best overall response; HR: hazard ratio; mPFS: median progression-free survival; CI: confidence interval; PFR: progression-free rate; PAN: panitumumab; BEV: bevacizumab; CET: cetuximab; IRI: irinotecan; ENCO: encorafenib; DER: deruxtecan; SOC: standard of care; SOTO: sotorasib; CMS: consensus molecular subtypes; ctDNA: circulating tumor DNA; BM: *BRAF* V600E-mutant; NE: not evaluated. Outcomes were expressed as HRs with 95% CIs or as median PFS with 95% CIs. Symbols (*, **, ***, ****, *****, &&, &&&, &&&&, &&&&&, $, $$) denote specific comparisons and do not indicate statistical significance. The only reported *p*-value is from Middleton et al. [82] (HR 4.33; *p* = 0.0012).

Patients with the G12C RAS mutation constitute roughly 3% of all mCRC patients. Data on the combination of G12C inhibitors and anti-EGFR agents has been reported in heavily pretreated mCRC patients (> 2 lines of therapy). Trials involving adagrasib, sotorasib, and divargasib in combination with anti-EGFR therapy have shown a BOR ranging from 26.4% to 62.5% and a median PFS of between 5.6 months and 8.1 months, both of which are clearly better than the SOC in this population (BOR < 5% and PFS of 3–4 months). Regardless, after an initial benefit, progression is typical, and fewer than 15% of patients remain progression-free at 12 months [72–74]. All of these drugs are now moving to first-line or second-line phase III trials in combination with doublets and compared with SOC, but data from these trials are not yet available. Finally, HER2-positive (+++) patients constitute less than 5% of mCRC patients. High activity in heavily pretreated patients (> 2 lines of therapy) has consistently been observed with trastuzumab deruxtecan (BOR, 28–45.3%) and trastuzumab/lapatinib (BOR, 38.1%) [75–77]. Again, despite this high activity, fewer than 15% of patients remain progression-free at 12 months. Trastuzumab/tucatinib plus FOLFOX is now being studied in a first-line phase III trial vs. SOC in HER2-positive double wild-type *RAS*/*BRAF* patients.

### Basal (intrinsic) mechanism of targeted therapy resistance

Anti-EGFR agents (cetuximab and panitumumab) have been used in combination with chemotherapy in patients with advanced CRC for 20 years. Well-known mechanisms of anti-EGFR resistance are mutations in *RAS* and, more recently, *BRAF*. Hyperselection for RAS and BRAF [evaluation of double wild-type in circulating tumor DNA (ctDNA)] increases the efficacy of anti-EGFR therapy vs. bevacizumab [67]. Although it appears that left-sided patients with double wild-type *RAS* and *BRAF* are more responsive to anti-EGFR agents than to bevacizumab [78], results remain contradictory. This may be due to biomarkers of anti-EGFR sensitivity being more highly expressed in left-sided tumors than in right-sided ones [79]. The CMS is the most widely tested signature for comparing efficacy between anti-EGFR compounds and bevacizumab in WT *RAS* patients. While an increased overall response rate was found with anti-EGFR therapy over bevacizumab in CMS2 and CMS4 subtypes in the FIRE-3 trial, no clear benefit in terms of PFS was seen with anti-EGFR over bevacizumab in the FIRE-3 and CALGB/SWOG 80405 clinical trials [80, 81]. Therefore, we can conclude that the CMS is not useful for defining treatment strategies in double wild-type mCRC.

The *BRAF* V600E-mutant (BM) transcriptional signature was developed as a potential biomarker for targeted therapies [doublet (anti-EGFR and anti-BRAF) or triplet (anti-EGFR, anti-BRAF, and anti-MEK)] in *BRAF* mutant patients. The main differences between the BM1 and BM2 subtypes are that BM1 defines a mesenchymal subtype with high immune infiltration, while BM2 is characterized by E2F and G2M cell cycle, OXPHOS metabolism, and low immune infiltration. The efficacy of doublet or triplet therapy based on the BM classification has been evaluated in three prospective clinical trials. The first trial evaluated the BM signature in heavily pretreated *BRAF* mutant patients receiving triplet therapy (dabrafenib, trametinib, and panitumumab). BM1 patients were found to benefit from triplet therapy [82]. Interestingly, this is the only study to collect tumor biopsies from metastases before therapy. Importantly, post-progression survival was shorter in BM2 subtype patients than in BM1 subtype patients. These results align with the data presented in the BEACON phase III trial, which showed that patients with a high cytotoxic signature (BM1 subtype) and treated with triplet therapy have better survival (median survival, ~10 months) than patients treated with doublet therapy or chemotherapy alone (median survival, ~6 months) [83].

Doublet therapy and SOC have been compared in two clinical trials [69, 83]. Contradictory results were presented regarding BM analysis. In Kopetz’s study [69], vemurafenib plus cetuximab exhibited greater benefit in BM1 subtype patients than in BM2 patients. In contrast, in the BEACON study, BM2 patients showed greater benefit from doublet therapy than from triplet therapy and SOC. Thus, we cannot conclude that the BM signature can currently discriminate strategies in BRAF patients treated with targeted agents (TAs). Because the BREAKWATER study was conducted with doublet therapy plus SOC vs. SOC alone in first-line therapy [71], BM signature data would provide critical insights for trials.

### Acquired mechanism of targeted therapy resistance

How the three metabolic subtypes of MSS CRC tumors defined by the IMMETCOLS signature dynamically adapt to targeted therapy pressure is currently unknown. Typically, tumor metastases that show a partial response to targeted therapy exhibit limited fluorodeoxyglucose and glutamine uptake in PET-CT [84]. Most studies evaluating the mechanisms of acquired resistance to TAs have been conducted using blood samples [83–87]. Common mechanisms of acquired resistance to these therapies are increased CIN [85] and mutational signatures related to defects in polymerase epsilon exonuclease repair and homologous recombination [83]. The rewiring mechanisms facilitating this common process (increased CIN and mutational signatures) for acquiring resistance after TA therapy are currently unclear.

A second mechanism of acquired resistance to TAs is related to the acquisition of mutations in the RAS, BRAF, EGFR, and MAP2K signaling pathways. Although new mutations in these pathways are associated with anti-EGFR, BRAF, and RAS (G12C) resistance, most are subclonal and thus have an unclear impact on resistance [72, 74, 83, 86]. In addition, acquired passenger mutations unrelated to RAS, BRAF, EGFR, and MAP2K signaling, as well as amplifications in cMET, MYC, FLT3, which frequently increase after TAs, have been reported by several groups [72, 74, 83]. Finally, Woolston et al. [87], in a small cohort of patients with acquired resistance to cetuximab, observed an increased TGF-β signature along with elevated tumor-infiltrating lymphocyte (TIL) infiltration.

In preclinical settings, a more complex metabolic mechanism of acquired targeted therapy resistance has been identified. Table 3 summarizes these mechanisms for each TA currently being evaluated in phase III trials for advanced CRC. Briefly, anti-EGFR therapy has been associated with the acquisition of EMT features (typical of our IMC1 subtype) in colorectal cell lines after cetuximab exposure [86], as well as in co-cultures of CAFs and lung cancer cell lines resistant to gefitinib or erlotinib [88]. This EMT shift has been suggested as a potential mechanism of anti-EGFR resistance. Interestingly, Apicella et al. [88] reported that lactate extrusion from cancer cells plays a critical role in NF-κB-mediated CAF activation. A metabolic adaptation, likely associated with our indolent IMC2 phenotype, has been described in persistent cells after selective pressure from TAs. This metabolic rewiring has been noted with anti-EGFR [89, 90], anti-BRAF [91, 92], anti-RAS [93], and anti-HER2 [94, 95] therapy and shown an increasing dependency on fatty acids (fatty acid oxidation) instead of the classic consumption of glucose or glutamine. In addition, the concomitant activation of multiple antioxidant pathways, such as ferroptosis [92] and the xCT [96], has been described. Finally, a well-documented mechanism of multiple anti-TA resistance that is likely related to our IMC3 subtype, such as CIN acquisition [83, 85, 86], has also been noted in preclinical studies with anti-EGFR and BRAF inhibition [97] and anti-RAS exposure [98].

**Table 3:** Acquired resistance biomarkers to targeted therapy.

**Study (*n*)**	**EGFR**	**BRAF**	**G12C**	**HER2**
Harrold et al. [85] (*n* = 52)*	CIN (2, 3)	CIN (2, 3)	CIN (2, 3)	CIN (2, 3)
Parseghian et al. [86] (*n* = 569)*	EMT (1) ctDNA (MS)	NE	NE	NE
Woolston et al. [87] (*n* = 15)*	EMT (1)	NE	NE	NE
Du et al. [89] (*n* = 78)**	SIRT5 (2)	NE	NE	NE
Van den Bossche et al. [90]**	FAO (2)	NE	NE	NE
Kopetz et al. [83] (*n* = 318)*	NE	ctDNA (MS)	NE	NE
Shen et al. [91] **	NE	FAO (2)	NE	NE
Yaeger et al. [72] (*n* = 25)*	NE	NE	ctDNA	NE
Desai et al. [74] (*n* = 14)	NE	NE	ctDNA	NE
Viale et al. [93]**	NE	NE	OXPHOS (FAO) (2)	NE
Salgueiro et al. [98]**	NE	NE	CIN, cMET amp (2, 3)	NE
Parida et al. [96]**	NE	NE	NE	xCT (2)
Feng et al. [94]**	NE	NE	NE	FAO (2)

CIN: chromosomal instability; MS: mutational signatures; ctDNA: circulating tumor DNA; amp: gene amplification; SIRT5: sirtuin 5; FAO: fatty acid oxidation; OXPHOS: oxidative phosphorylation; EMT: epithelial-mesenchymal transition; IMMETCOLS: immune-metabolic signature; xCT: cystine/glutamate antiporter (mechanism of ferroptosis resistance); NE: not evaluated. IMC1 (1), IMC2 (2), IMC3 (3): metabolic subtypes according to the IMMETCOLS classification. *: Clinical studies; **: preclinical studies. Most acquired mutations in RAS, BRAF, EGFR, and MAP2K1 were subclonal.

## Conclusions

Understanding acquired resistance to chemotherapy and targeted therapy may help to guide subsequent treatment strategies. In this review, we emphasize that, despite some complexities, each of the three IMMETCOLS subtypes adapts its metabolism to overcome initial treatment sensitivity. In addition, all three subtypes can reduce the production of lethal ROS by enhancing antioxidant defenses in cancer cells (IMC2 and IMC3 subtypes) or by modulating redox signaling in CAFs (IMC1 subtype). Although IMMETCOLS has not yet been validated for routine clinical use, its classification may inform the development subtypes-specific strategies aimed at delaying or preventing acquired resistance. Therefore, we propose two translational perspectives. First, new strategies to overcome acquired resistance should be tailored to each of these subtypes rather than applied uniformly across all patients. Second, these strategies should be designed to target specific vulnerabilities that emerge under treatment pressure.

## Abbreviations

BM: *BRAF* V600E-mutant
BOR: best overall response
CAF: cancer-associated fibroblast
CI: confidence interval
CIN: chromosomal instability
CMS: consensus molecular subtypes
ctDNA: circulating tumor DNA
EMT: epithelial-mesenchymal transition
G6PD: glucose-6-phosphate dehydrogenase
GSH: glutathione
HR: hazard ratio
iCAFs: inflammatory cancer-associated fibroblasts
IMMETCOLS: immune-metabolic signature
LIF: leukemia inhibitory factor
mCAFs: myofibroblast cancer-associated fibroblasts
mCRC: metastatic colorectal cancer
MRE11: meiotic recombination 11 homolog
MSI: microsatellite-unstable
MSS: microsatellite-stable
ODC1: ornithine decarboxylase 1
OXPHOS: oxidative phosphorylation
PDS: pathway-derived subtype
PLK1: polo-like kinase 1
PPP: pentose phosphate pathway
ROS: reactive oxygen species
SAT1: N1-acetyltransferase
scRNA: single-cell RNA
SIRT1: sirtuin 1
SOC: standard of care
TAs: targeted agents
TCA: tricarboxylic acid cycle
TGF-β: transforming growth factor-β
TIL: tumor-infiltrating lymphocyte
TKT: transketolase
TNBC: triple-negative breast cancer
xCT: cystine/glutamate antiporter
α-SMA: α-smooth muscle actin

## Declarations

### Availability of data and materials

Both figures were created de novo by the authors using BioRender.com and do not reproduce or adapt material from previously published sources.

### Copyright

© The Author(s) 2025.
